# Niclosamide suppresses renal cell carcinoma by inhibiting Wnt/β-catenin and inducing mitochondrial dysfunctions

**DOI:** 10.1186/s40064-016-3153-x

**Published:** 2016-08-30

**Authors:** Juan Zhao, Qiushan He, Zhimin Gong, Sen Chen, Long Cui

**Affiliations:** 1 Department of Oncology, Xiangyang Central Hospital, The Affiliated Hospital of Hubei College of Arts and Science, Xiangyang, 441021 People’s Republic of China; 2 Department of Academic Affairs, Hubei University of Medicine, Shiyan, 441021 People’s Republic of China; 3 Department of Nephrology, Xiangyang Central Hospital, The Affiliated Hospital of Hubei College of Arts and Science, 39 Jingzhou Street, Xiangyang, 441021 People’s Republic of China

**Keywords:** Renal cell carcinoma, Niclosamide, Wnt/β-catenin, Mitochondria

## Abstract

**Purpose:**

To investigate the effects of anthelminthic drug niclosamide in renal cell carcinoma (RCC) and the underlying mechanisms of its action.

**Methods:**

The effects of niclosamide on the proliferation and apoptosis of RCC cells were examined in vitro and in vivo by using MTS, colony formation assay, flow cytometry and xenograft cancer mouse model. Mechanism studies were performed by analyzing Wnt/β-catenin signaling and mitochondrial functions in a panel of RCC cell lines.

**Results:**

We show that niclosamide effectively targets two RCC cell lines through inhibiting proliferation and anchorage-independent colony formation, and inducing apoptosis. It also enhances the inhibitory effects of chemotherapeutic drug cisplatin in two independent in vivo RCC xenograft mouse models. Mechanistically, niclosamide decreases β-catenin levels and therefore suppresses Wnt/β-catenin activities. Overexpression of β-catenin partially reverses the inhibitory effects of niclosamide in RCC cells, demonstrating that besides β-catenin, other mechanisms are involved in niclosamide’s anti-cancer activity. Indeed, we further show that niclosamide induces mitochondrial dysfunctions as shown by the decreased level of mitochondrial membrane potential and respiration, resulting in decreased ATP levels and increased reactive oxygen species (ROS) levels.

**Conclusions:**

Our findings support the inhibitory effects of niclosamide in cancer and provide better understanding on its underlying mechanism. Our data suggests that niclosamide is a useful addition to the treatment armamentarium for RCC.

## Background

Renal cell carcinoma (RCC) is an epithelial tumour derived from the proximal tubules of nephrons (Thoenes et al. 1986). It is the most aggressive type of genitourinary cancer and resistant to chemotherapy, radiotherapy and targeted therapy (Bukowski 1997; Kanesvaran and Tan 2014). The molecular mechanisms leading to RCC development, progression and resistance are complex and involve abnormal genetic modifications and aberrant activation of oncogenic signaling pathways, such as Wnt/β-catenin, phosphatidylinositol 3-kinases (PI3 K)/Akt and hepatocyte growth factor (HGF)/c-MET pathways (Zhou et al. 2015; Cojocaru et al. 2015). The identification of therapeutic agents that targets these oncogenic pathways may have the potential to improve the efficacy for RCC treatment.

Niclosamide is an anthelmintic drug especially used for the treatment of cestodes infection (Tanowitz et al. 1993). It kills parasites through inhibiting mitochondrial oxidative phosphorylation (Weinbach and Garbus 1969). However, niclosamide has been recently identified as a novel type of anti-cancer drug. It suppresses growth of several tumor cell lines (Arend et al. 2014; Osada et al. 2011; Lu et al. 2011; Khanim et al. 2011). When niclosamide is combined with conventional chemotherapeutic drugs, the combination showed significant enhanced anti-cancer effects (Liu et al. 2016). Several work highlight niclosamide as a potent Wnt/β-catenin inhibitor and that inhibition of β-catenin signalling is the mechanism of its action on cancer (Arend et al. 2014; Osada et al. 2011; Lu et al. 2011; Chowdhury et al. 2016). Others believe that niclosamide’s anti-cancer activity is mediated through mitochondria, NF-κB pathway or androgen receptor signalling (Khanim et al. 2011; Liu et al. 2016; Jin et al. 2010). Nevertheless, the molecular mechanisms of niclosamide in cancer are not well defined.

The potent inhibitory effects on a panel of tumor cell lines and its known cytotoxicity as well as pharmacokinetic profiles make niclosamide an attractive candidate for cancer treatment. Although its anti-tumor activities had been reported in various tumors, the effectiveness of niclosamide in RCC is unknown. In this study, we investigated the effects of niclosamide and its underlying mechanisms in RCC cells using in vitro cellular culture system and in vivo xenograft mouse tumor model. To further explore the application potential of niclosamide in RCC, the combinatory effects of niclosamide and chemotherapeutic drugs were examined.

## Methods

### Cells and drugs

Human renal carcinoma cell lines, A-498 and SW-839 were purchased from American Type Culture Collection. Cells were cultured in Eagle’s Minimal Essential Media (MEM) supplemented with a final concentration of 10 % fetal bovine serum (Hyclone, UK) and 100 u/ml penicillin–streptomycin (Invitrogen, US). Niclosamide (BioVision Inc. US) and cisplatin (Sigma, US) were dissolved in DMSO and water, respectively.

### MTS proliferation assay

Cells at ten thousands were seeded in 96 well plates overnight. The next day, cells were treated with niclosamide for 72 h. 20 µl of tetrazolium compound [3-(4,5-dimethylthiazol-2-yl)-5-(3-carboxymethoxyphenyl)-2-(4-sulfophenyl)-2H-tetrazolium, inner salt; MTS] was added to each well and incubated for 2 h. The absorbance was measured at 490 nm.

### Annexin V flow cytometry analysis

Cells at 1 × 10^5^ were seeded at 12 well plate and treated with niclosamide for 72 h. After treatment, cells were detached using trypsin (Invitrogen) and stained with Annexin V-FITC (BD Pharmingen, US). The stained cells were analysed by flow cytometry on a Beckman Coulter FC500 and a minimum of 5000 events were counted. The percentage of Annexin V-positive cells was determined by CXP software.

### Colony formation assay

A bottom layer of a final concentrating of 0.7 % Bacto agar was prepared in 12 well plates. Cells at 1000 together with niclosamide were seeded in a plate with a top layer of 0.3 % Bacto agar. 100 µl of culture medium was added to the top layer and replaced with fresh one twice a week. After 10–14 days, the colonies were stained with crystal violet and the number of colonies were scored.

### Western blot (WB) analyses

Total protein were lysed by using radioimmunoprecipitation assay (RIPA) buffer (Invitrogen). Equal amount of proteins were resolved using denaturing SDS–PAGE and analyzed by WB using antibodies recognizing β-catenin and β-actin (Santa Cruz Biotechnology Inc, US).

### Mitochondrial metabolic assay

Cells were treated with different concentrations of niclosamide for 24 h prior to mitochondrial function analysis. To measure mitochondrial membrane potential, cells were stained with 5,5′,6,6′-tetrachloro-1,1′,3,3′-tetraethyl benzimidazolylcarbocyanine iodide (JC-1, Invitrogen) and analysed by flow cytometry on a Beckman Coulter FC500. Oxygen Consumption Rate (OCR) was measured using a Seahorse XF24 extracellular flux analyser (Seahorse Bioscience, US) according to manufacturer’s instructions. Analyses were performed at basal conditions. ATP levels were measured by ATPlite Luminiescent Assay kit (Perkin Elmer, US) according to the manufacturer’s protocol. To measure intracellular ROS, cells were incubated with 10 µM CM-H_2_DCFDA (Invitrogen) and absorbance at ex/em of 495/525 nm were measured.

### Plasmid transfection

Transfections were carried out in A-498 and SW-839 cells by using nucleofection (Lonza, US). The M50 Super 8× TOPFlash plasmid (a kind gift from Dr. Randall Moon) (Veeman et al. 2003) and Luciferase Reporter Assay System (Promega, US) were used for TOPflash assay. For β-catenin overexpression, cells were transfected with 1.5 μg pcDNA, or pcDNA-β-cat (human β-catenin pcDNA3 plasmid, a kind gift from Dr. Eric Fearon) (Kolligs et al. 1999). At 24 h after transfection, cells were used to perform WB or rescue experiments.

### RCC xenograft in SCID mouse

The animal experiments were approved by the Institutional Animal Care and Use Committee of Xiangyang Central Hospital. SCID mice (Biocytogen Inc, China) were subcutaneously injected with 100 µl mixture (1:1) of 10 million cells and Matrigel (BD Biosciences, US). The mice were treated with vehicle control, intraperitoneal niclosamide at 10 mg/kg, cisplatin at 50 mg/kg or combination of both. Tumour volume were measured every three days.

### Statistical analyses

The data are expressed as mean and standard deviation. Statistical analyses were performed by unpaired Student’s t test. p value <0.05 were considered statistically significant.

## Results

### Niclosamide is active against renal carcinoma cells

**Fig. 1:**
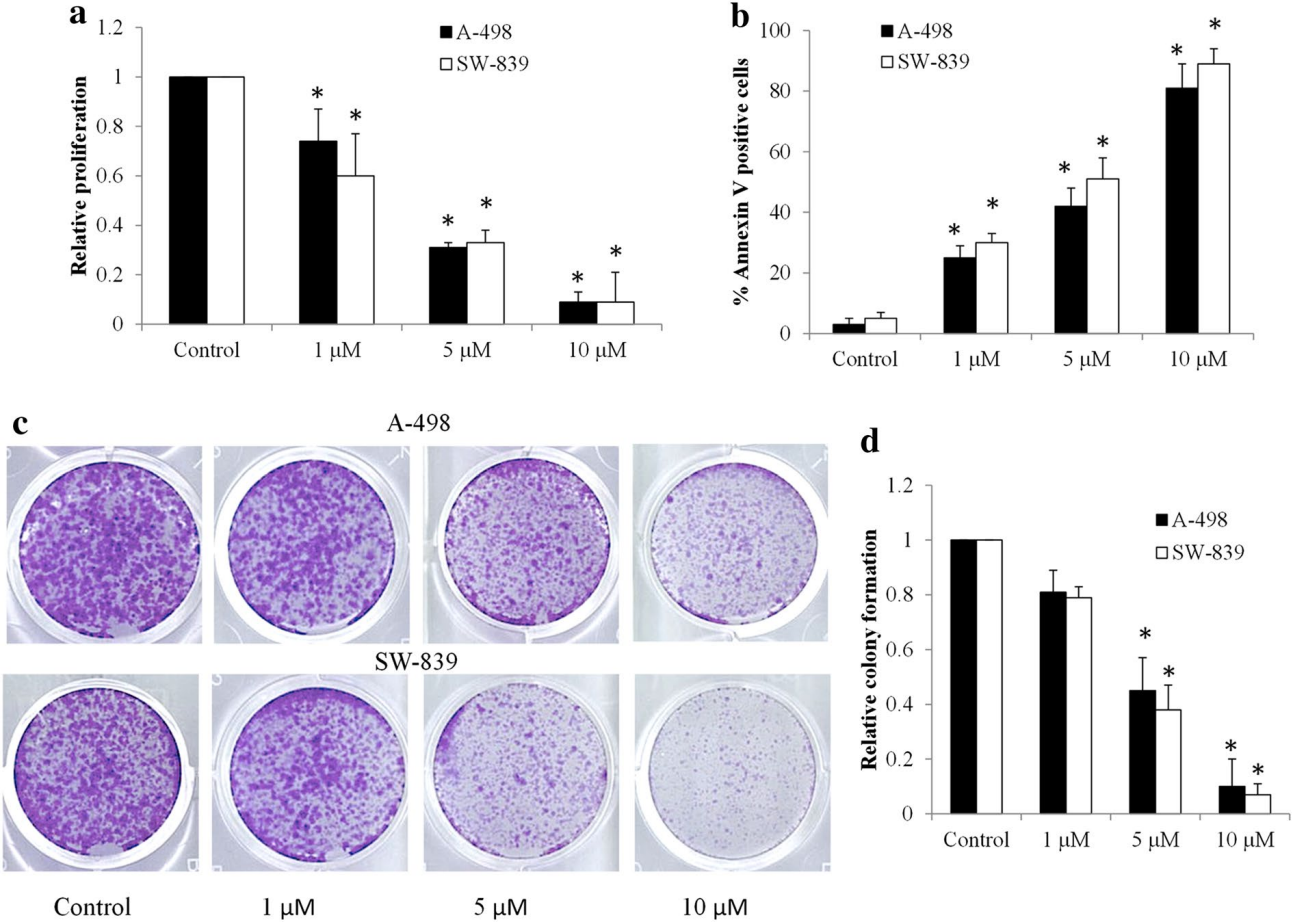
Niclosamide inhibits proliferation and anchorage-independent colony formation and induces apoptosis in RCC cell lines. **a** Niclosamide decreases proliferation of A-498 and SW-839 cells. **b** Niclosamide induces apoptosis of A-498 and SW-839. **c** Representative images taken at 14 days of an anchorage-independent colony formation assay. **d** Quantification of colonies show the dose-dependent inhibitory effect of niclosamide on colony formation in RCC cells. *p < 0.05, compared to control

Two human RCC cell lines, A-498 and SW-839, were used in this study. These cell lines were treated with various concentrations of niclosamide for 3 days prior to MTS and Annexin V analysis. We found that niclosamide significantly inhibited proliferation in a dose-dependent manner in A-498 and SW-839 cells (Fig. 1a). In addition, niclosamide dose-dependently induced apoptosis of these two cell lines (Fig. 1b). Anchorage-independent growth in soft agar is often predictive of tumorigenicity as colonies formed in soft agar are from cells displaying both highly proliferative and invasive phenotypes (Gao et al. 2005). We found that niclosamide was also effective in inhibiting anchorage-independent growth of RCC cells (Fig. 1c, d). The IC_50_ of niclosamide on A-498 is similar to the one in SW-839, suggesting that niclosamide is active against RCC cells regardless of their cellular origin.

### The inhibitory effects of niclosamide on renal carcinoma cells are partially through suppressing Wnt/β-catenin pathway

**Fig. 2:**
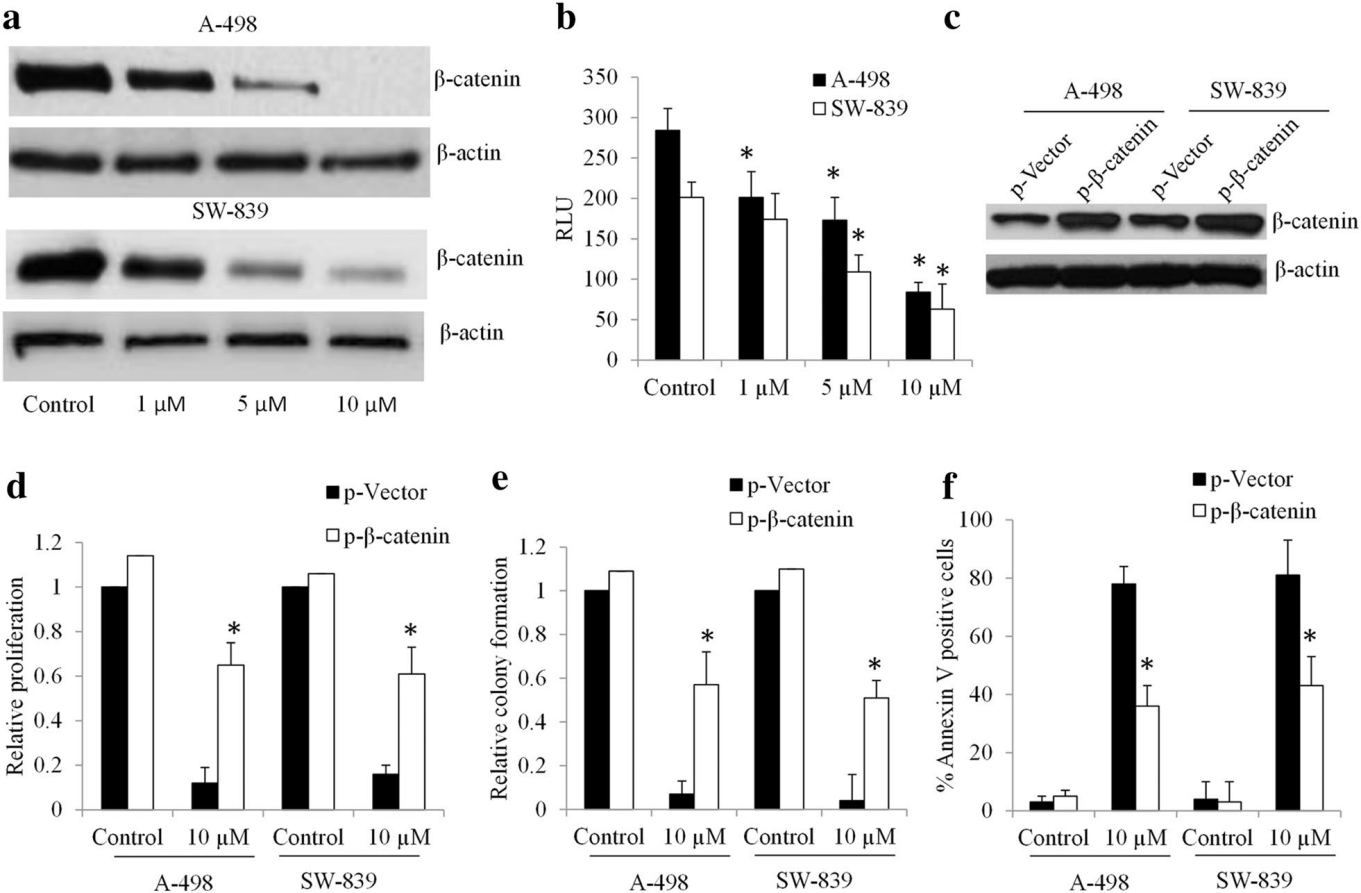
Niclosamide inhibits Wnt/β-catenin signaling in RCC cells. **a** Niclosamide decreases intracellular β-catenin levels in A-498 and SW-839 cells. Cells were treated with niclosamide for 24 h prior to WB analysis. **b** Niclosamide inhibits TOPflash activation. RCC cells transfected with TOPflash plasmid were treated with niclosamide as indicated. RLU, relative light units. **c** β-Catenin level is increased n A-498 and SW-839 cells transfected with β-catenin overexpression plasmid. Overexpression of β-catenin partially rescued the effects of niclosamide in inhibiting proliferation (**d**) and colony formation (**e**) and inducing apoptosis (**f**) in RCC cells. *p < 0.05, compared to control or p-Vector

Several studies have demonstrated the potent inhibitory effects of niclosamide on Wnt/β-catenin signaling pathway in various types of cancers (Arend et al. 2014; Osada et al. 2011; Lu et al. 2011). Consistent with these findings, our work demonstrated that niclosamide decreased levels of intracellular β-catenin in RCC cells (Fig. 2a). To confirm the inhibitory effect of niclosamide on Wnt/β-catenin signaling in RCC cells, we performed Wnt/β-catenin reporter assay by transiently transfected with TOPFlash plasmid. Niclosamide dose-dependently inhibited the TOPFlash activity in RCC cells (Fig. 2b), which correlates well with the decreased levels of β-catenin in RCC cells exposed to niclosamide (Fig. 2a).

To investigate whether β-catenin stabilization can abolish the effects of niclosamide, we rescued β-catenin levels by overexpressing β-catenin in RCC cells (Fig. 2c). In β-catenin-overexpressing A-498 and SW-839 cells, niclosamide is less effective in inhibiting proliferation and colony formation, and inducing apoptosis compared to control cells (Fig. 2d–f). These data demonstrate that inhibition of Wnt/β-catenin signalling is the molecular mechanism of niclosamide’s action in RCC cells. It is also noted that β-catenin-overexpression failed to completely rescue the inhibitory effects of niclosamide, suggesting that other mechanisms are involved for the observed biological effects of niclosamide in RCC.

### Niclosamide induces mitochondrial dysfunctions

It is known that niclosamide kills the tapeworm through inhibiting oxidative phosphorylation (Weinbach and Garbus 1969). Khanim et al. recently demonstrated that niclosamide targets mitochondria in multiple myeloma cells by reducing free light chain production (Khanim et al. 2011). We therefore investigated the effects of niclosamide on mitochondrial functions in RCC cells.

**Fig. 3:**
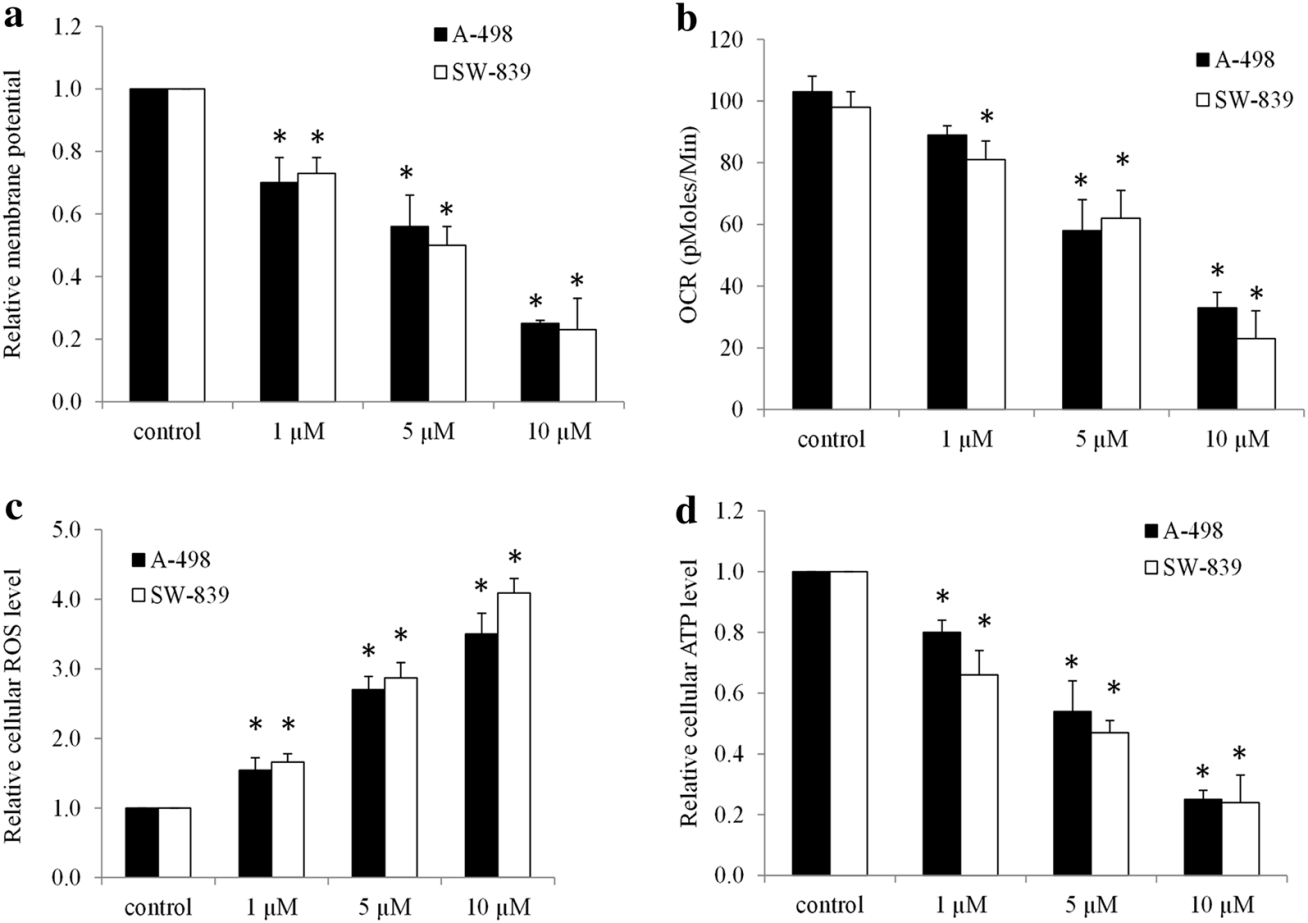
Niclosamide impairs mitochondrial function and induces oxidative stress. Niclosamide decreases mitochondrial membrane potential (**a**), inhibits mitochondrial respiration (**b**), increases ROS levels (**c**) and reduces ATP levels (**d**) in RCC cells. *p < 0.05, compared to control

We found that niclosamide decreased mitochondrial membrane potential at the same effective concentrations (e.g., 1, 5, and 10 µM) that inhibits proliferation and induces apoptosis (Fig. 3a). In addition, niclosamide significantly inhibited OCR, decreased ATP levels and increased ROS levels in RCC cells (Fig. 3b–d). Taken together these data show that niclosamide impairs mitochondrial functions to induce an energy crisis and oxidative damage in RCC cells.

### Niclosamide inhibits growth of renal carcinoma in vivo and enhances the effects of chemotherapeutic drug

**Fig. 4:**
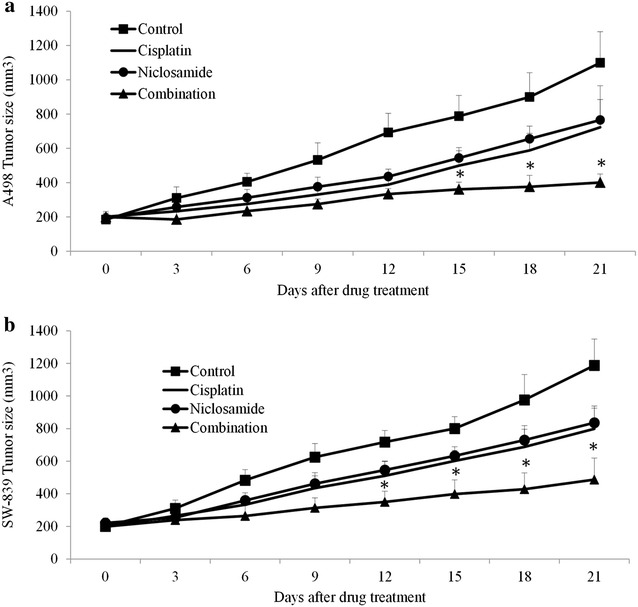
Niclosamide inhibits RCC tumor growth and enhances the inhibitory effect of cisplatin in vivo. Niclosamide inhibited growth of RCC tumors derived from A-498 (**a**) and SW-839 (**b**) cells as a single drug. Combination of niclosamide and cisplatin significantly inhibited much more tumor growth throughout the duration of treatment in two xenograft RCC models. *p < 0.05, compared to control or single arm

We next tested the in vivo efficacy of niclosamide and investigated whether its combination with cisplatin (common chemotherapeutic drug) resulted in greater efficacy than single drug alone. We established two RCC xenograft mouse models by subcutaneous injection of A-498 or SW-839 cells into nude mice. When mice tumor reached 200 mm^3^, mice were treated with vehicle control, intraperitoneal niclosamide, cisplatin or combination of niclosamide and cisplatin for 3 weeks. No significant body weight loss and abnormal behavior in all groups (data not shown) indicate that the mice tolerated the treatment well. We further show that niclosamide inhibited growth of tumors derived from both A-498 and SW-839 cells (Fig. 4). It is noted that niclosamide at 10 mg/kg achieved similar efficacy as chemotherapeutic drug cisplatin at 40 mg/kg. More importantly, the combination of niclosamide and cisplatin further inhibited tumor growth in both RCC xenograft models (Fig. 4). Our in vivo data are consistent with in vitro data, and confirmed the anti-cancer activities of niclosamide in RCC.

## Discussion

Current therapies including chemotherapy, radiotherapy and targeted therapy have not yielded durable remissions in RCC patients (Kanesvaran and Tan 2014; Lisa et al. 2016). There are many ongoing research efforts to discover drugs to eradicate RCC cells. The discovery, development and registration of novel compounds can be a long and laborious process. An alternative to new drug development is drug repositioning. For example, thalidomide has been successfully repurposed for myeloma treatment (Mutsaers 2007; Chong and Sullivan 2007). In this study, we evaluated anthelmintic drug niclosamide as a potential candidate for RCC treatment. We are the first to demonstrate the suppression of Wnt/β-catenin and induction of mitochondrial dysfunctions in RCC cells by niclosamide, leading to proliferation inhibition and cell death.

Two cell lines, A-498 and SW-839, selected for demonstration of the biological effects of niclosamide are derived from the most frequent clear-cell tumor subtype of RCC (accounting for >80 % of all RCCs) (Kovacs et al. 1997). Our study show that niclosamide significantly inhibits proliferation and induces apoptosis in both A-498 and SW-839 cells (Fig. 1a, b), suggesting the therapeutic efficacy of niclosamide in the majority of RCCs. Niclosamide also significantly inhibits anchorage-independent colony formation of RCC cells (Fig. 1c, d), further demonstrating its inhibitory effects on RCC subpopulations with highly proliferative and invasive properties (Gao et al. 2005). The efficacy of niclosamide have been demonstrated in various cancers, such as colon, cancer, ovarian cancers and myeloma (Arend et al. 2014; Osada et al. 2011; Khanim et al. 2011). Our data supports the previous studies on the inhibitory effects of niclosamide in cancer and adds RCC to the growing list of niclosamide-targeted cancers.

Consistent with the in vitro data, niclosamide inhibits growth of two independent RCC cancer xenograft mouse models established by using A-493 and SW-839 cells (Fig. 4), confirming its in vivo efficacy. Notably, niclosamide further enhanced the inhibitory effect of cisplatin in RCC tumor growth (Fig. 4), demonstrating the synergism of niclosamide and chemotherapeutic drugs in RCC. In addition, our data show that mice tolerate very well to niclosamide at 10 mg/kg given by intraperitoneal injection (data not shown). Although niclosamide has little absorption from gastrointestinal tract, pharmacokinetic analysis of niclosamide following oral administration in mice by Osada et al. show the efficient and reliable destruction of niclosamide from blood to tumor tissue (Osada et al. 2011). Our data together with previous studies suggest that greater absorption of niclosamide can be achieved either by drug modifications or by using different administration route.

The mechanism of action of niclosamide seems to be in a cancer cell-type specific manner. Some have reported that niclosamide suppresses Wnt/β-catenin signalling via inducing lipoprotein receptor-related protein-6 (LRP6) degradation in prostate and breast cancer cells (Lu et al. 2011). Others have demonstrated that niclosamide induces free light chain production in myeloma cells (Khanim et al. 2011). We found that niclosamide decreased levels of β-catenin and therefore suppressed Wnt/β-catenin activities in A-498 and SW-839 cells (Fig. 2a, b). Furthermore, overexpression of β-catenin levels partially abolished the inhibitory effects of niclosamide in RCC cells (Fig. 2c–f). These data agree with the previous work that niclosamide targets RCC cells through inhibiting Wnt/β-catenin signalling pathways. Aberrant Wnt/β-catenin signalling pathway caused by APC deficiency or HIF1α play important roles in RCC progression (Cojocaru et al. 2015; Majid et al. 2012). Wnt/β-catenin signalling pathway has been the target of major drug discovery programs in the past decade. In line with these efforts, our work agrees with the essential roles of Wnt/β-catenin in RCC and further demonstrates that it can be effectively targeted by niclosamide.

However, the partial but not complete rescue by β-catenin overexpression suggests that other mechanisms of action might be involved in the cells treated with niclosamide. Indeed, we found that niclosamide induces mitochondrial dysfunctions via decreasing mitochondrial membrane potential, OCR and ATP levels (Fig. 3a, b, d). A consequence of impaired mitochondrial function induced by niclosamide is the increased oxidative damage as shown by the increased levels of ROS (Fig. 3c). Targeting cancer metabolism, such as mitochondrial metabolism, has recently become an attractive therapeutic strategy in cancer due to their unique dependence on oxidative phosphorylation (Jaras and Ebert 2011; Loureiro et al. 2013; Moreno-Sanchez et al. 2007; Funes et al. 2007). Various antibiotics or anthelminthic drugs that target mitochondria have been reported to be effective in inhibiting a large panel of cancer cells (Lamb et al. 2015). Our work together with the previous work suggest that induction of mitochondria dysfunctions might be a common mechanism of the action of those antibiotics in cancers.

In conclusion, our results show that niclosamide effectively targets RCC cells through both suppressing Wnt/β-catenin signalling and inducing mitochondrial dysfunctions. Importantly, niclosamide significantly enhances the effect of chemotherapeutic drug cisplatin in inhibiting tumour growth in two-independent RCC xenograft models. Our findings suggest that niclosamide may have utility in treating patients with RCC.
